# Antibodies to Influenza A(H5N1) Virus in Hunting Dogs Retrieving Wild Fowl, Washington, USA

**DOI:** 10.3201/eid3006.231459

**Published:** 2024-06

**Authors:** Justin D. Brown, Adam Black, Katherine H. Haman, Diego G. Diel, Vickie E. Ramirez, Rachel S. Ziejka, Hannah T. Fenelon, Peter M. Rabinowitz, Lila Stevens, Rebecca Poulson, David E. Stallknecht

**Affiliations:** Pennsylvania State University, University Park, Pennsylvania, USA (J.D. Brown, L. Stevens);; Adam Black Veterinary Services, Anacortes, Washington, USA (A. Black);; Washington Department of Fish and Wildlife, Olympia, Washington, USA (K.H. Haman);; Cornell University College of Veterinary Medicine, Ithaca, New York, USA (D.G. Diel);; University of Washington, Seattle, Washington, USA (V.E. Ramirez, R.S. Ziejka, H.T. Fenelon, P.M. Rabinowitz);; University of Georgia College of Veterinary Medicine, Athens, Georgia, USA (R. Poulson, D.E. Stallknecht)

**Keywords:** influenza, H5N1, highly pathogenic, avian influenza virus, viruses, hunting dogs, retriever dogs, Washington, United States, respiratory infections, zoonoses

## Abstract

We detected antibodies to H5 and N1 subtype influenza A viruses in 4/194 (2%) dogs from Washington, USA, that hunted or engaged in hunt tests and training with wild birds. Historical data provided by dog owners showed seropositive dogs had high levels of exposure to waterfowl.

Since 1996, goose/Guangdong lineage H5 highly pathogenic influenza A viruses (HPIAV) have caused an unprecedented panzootic. In 2020, subclade 2.3.4.4b HPIAV H5N1 emerged and spread to multiple continents causing substantial death in poultry and wild birds. Recently, increased detection in mammals has stimulated concern that the virus might be adapting to mammal hosts.

Despite the prolonged global epizootic of HPIAV H5N1, reported infections in dogs have been rare. During an HPIAV H5N1 outbreak in Thailand, a fatal canine infection in 2004 associated with a dog eating a duck carcass was reported (*1*). A follow-up serosurvey of outwardly healthy stray dogs in Thailand detected HPIAV H5N1 antibodies in 25.4% (160/629) of sampled dogs (*2*). During April 2023, another fatal HPIAV H5N1 infection was identified in Ontario, Canada, in a dog that developed severe respiratory and systemic signs shortly after chewing on a dead wild goose (*3*). In experiments, beagles were susceptible to HPIAV H5N1 infections, during which some infected dogs excreted high concentrations of virus through the respiratory tract and experienced severe disease (*4*). In contrast, previous studies in beagles reported susceptibility to HPIAV H5N1 infection that manifested with moderate to no clinical signs (*5*,*6*). Existing field and experimental data collectively suggest dogs are susceptible to HPIAV H5N1 infection but clinical outcomes vary. However, infection appears to be restricted to dogs with high virus exposure. To investigate this further, we tested for antibodies to influenza A(H5N1) virus in bird hunting dogs, a category of dogs at high risk for contact with HPIAV H5N1–infected wild birds, and compared serologic results to reported hunting or training activities.

Dog owners completed a questionnaire providing details about their dogs’ retrieving activities, canine influenza virus (CIV) vaccination status, and clinical history. Methods used in this research were approved by the Institutional Animal Care and Use Committee at Penn State University (#202302394).

## The Study

During March–June 2023 in Washington, USA, we collected blood samples from 194 dogs identified by owners as having engaged in bird hunting or bird hunt tests and training over the previous 12 months (Figure). Waterfowl hunting season in Washington extends from mid-October through February; consequently, we collected samples 1–4 months after season closure. We collected blood from the jugular vein, immediately centrifuged it, and stored it at 4ºC in the field, then stored serum at –20°C until testing was performed. 

**Figure:**
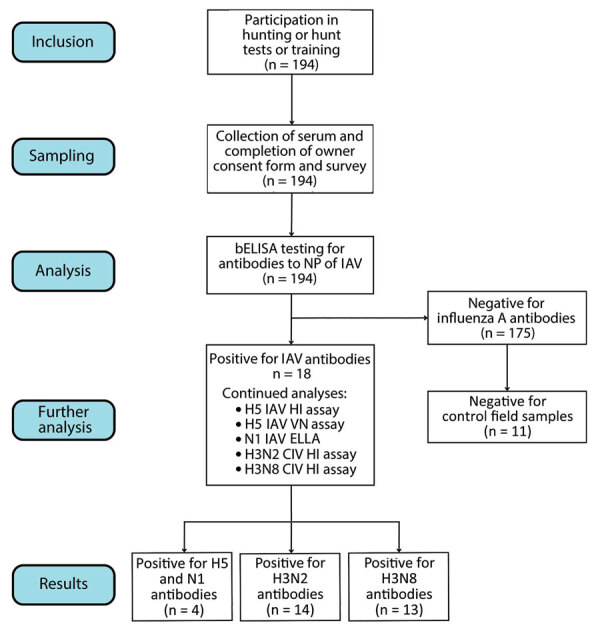
Flow diagram of participation in a serosurvey for antibodies to IAV in hunters and their hunting dogs, Washington, USA. CIV, canine influenza virus; ELLA, enzyme-linked lectin assay; HI, hemagglutination inhibition; IAV, influenza A virus; NP, nucleoprotein; VN, virus neutralization

We screened serum samples for antibodies to influenza A virus (IAV) nucleoprotein using an MultiS-Screen Ab blocking ELISA (bELISA; IDEXX, https://www.idexx.com), according to manufacturer instructions. We subsequently tested all bELISA-positive samples for antibodies to H5 using a hemagglutination inhibition (HI) assay and virus neutralization (VN), and to N1 with an enzyme-linked lectin assay (ELLA) using published protocols (*7*,*8*). In addition, we ran 11 bELISA-negative samples as negative field controls. In both the HI and VN assays, we used 2 reverse genetics antigens to detect antibodies to clade 2.3.4.4b H5 and North American H5 low pathogenic IAV. Antigens included IDCDC-RG71A containing Eurasia hemagglutinin (HA) and neuraminidase (NA) from A/Astrakhan/3212/2020(H5N8) on an A/Puerto Rico/8/1934(H1N1)(PR8) backbone and LP-RGBWT/TX that included North America HA and NA from A/blue-winged teal/AI12–4150/Texas/2012(H5N2) on a PR8 backbone. The HA of IDCDC-RG71A has a modified protease cleavage site consistent with a low pathogenic IAV phenotype. For the ELLA, we used A/ruddy turnstone/New Jersey/AI13-2948/2013(H10N1) as the antigen. 

We used conservative positive threshold titers: H5 HI, >1:32; H5 VN, >1:20; and N1 ELLA, >1:80. We considered samples H5 seropositive if positive for H5 using HI assay or VN and N1 seropositive if positive for N1 using ELLA. We also tested all bELISA-positive serum samples for antibodies to H3N2 and H3N8 CIV by HI assay (positive threshold ≥1:8) (*9*). We calculated seroprevalence using R (*10*). 

Most dogs retrieved waterfowl (86%), and many (69%) retrieved both waterfowl and upland game birds (Appendix Tables 1, 2). Dogs most commonly contacted dabbling ducks (81% of dogs), which are notable reservoirs for HPIAV H5N1. Dogs also frequently contacted birds from other categories considered high risk for HPIAV H5N1, including geese (32% of dogs) and diving ducks (23% of dogs) (Appendix Table 3). Most dogs had retrieved or trained multiple times during the previous 12 months; 38% were reported to have been in the field during ≥15 hunts and 78% reported to have trained with live or dead birds ≥15 times (Appendix Table 2). Reportedly 11% of dogs retrieved dead or clinically ill waterfowl that showed no evidence of having been shot or hunted. 

Antibodies to IAV were detected by bELISA in 18/194 (9.3%, 95% CI 5.6%–14.3%) dogs not displaying overt disease. Of the 18 bELISA-positive samples, 14 (77.8%, 95% CI 52.4%–93.6%) were seropositive for CIV H3N2 and 13 (72.2%, 95% CI 46.5%–90.3%) for CIV H3N8. The closeness of those results might have resulted from cross-reactivity between CIV H3N2 and H3N8. Of the 18 bELISA-positive dogs, 12 (66.7%, 95% CI 41.0%–86.7%) had reportedly been vaccinated for CIV. Four (22%, 95% CI 6.4%–47.6%) of the 18 were seropositive for H5 antibodies when tested using HI or VN and for N1 antibodies when tested using ELLA (Table). Two dogs were seropositive for H5 by HI and 3 dogs by VN, both using the IDCDC-RG71A antigen. No dogs had antibodies detectable above the positive threshold for H5 using the North America LP-RGBWT/TX antigen. Despite using an unmatched N1, all 4 H5-seropositive dogs tested positive for N1 antibodies using ELLA. Three of the H5- and N1-seropositive dogs had not been vaccinated for CIV and were negative for H3N2 and H3N8 antibodies. One H5- and N1-seropositive dog reportedly had been vaccinated in 2021 and had a low antibody titer to CIV H3N2 (1:8). All 11 bELISA-negative serum samples (i.e., negative controls) tested negative on HI and VN for antibodies to H5; however, 3 were seropositive for N1. The cause of the N1 seropositivity is unknown; however, serologic evidence of pandemic H1N1 infections in dogs has been previously reported, and additional testing is warranted (*11*). 

**Table:** Data for individual hunting dogs that tested positive for antibodies to H5 and N1 influenza A virus, Washington, USA*

Dog ID	Mean S/N	Influenza A subtype-specific test results					
		HI rg BWT	HI rg AST H5 (titer)	VN rg AST H5	ELLA N1	HI-CIV H3N2	HI-CIV H3N8
HD23-21	0.275	Neg	+ (32)	–	+ (320)	+ (8)	–
HD23-30	0.345	Neg	–	+ (80)	+ (>2,560)	–	–
HD23-135	0.474	Neg	–	+ (40)	+ (>2,560)	–	–
HD23-139	0.375	Neg	+ (1:32)	+ (320)	+ (>1,280)	–	–

*Determined using AI MultiS-Screen AB blocking ELISA (IDEXX Laboratories, https://www.idexx.com), HI assay, virus neutralization, and ELLA. Positive threshold titers for the influenza A subtype-specific tests: H5 HI, >1:32; H5 VN, >1:20; N1 ELLA, >1:80; H3N2 and H3N8 CIV HI assay, ≥1:8. Negative antibody results based on the reported positive threshold titer for each assay. BWT, wild-type (wt) influenza B virus; CIV, canine influenza virus; ELLA, enzyme-linked lectin assay; HI, hemagglutination inhibition; ID, identifier; rg, reverse genetics; N1, A/ruddy turnstone/New Jersey/AI13–2948/2013(H10N1) antigen; rg AST, modified HA and NA antigen from A/Astrakhan/3212/2020(H5N8); rg BWT, HA and NA antigen from A/blue-winged teal/AI12-4150/Texas/2012(H5N2); S/N, ratio of sample to negative control absorbance values; VN, virus neutralization; –, negative antibody result.

Over the previous 12 months, all 4 H5- and N1-seropositive dogs reportedly had hunted waterfowl extensively in areas affected by H5N1 HPIAV outbreaks in wild waterfowl. Three H5- and N1-seropositive dogs reportedly had retrieved waterfowl that were either dead or had neurologic symptoms but that showed no evidence of having been shot or hunted. Two H5- and N1-seropositive dogs were from households that owned multiple hunting dogs included in this study; 1 seropositive dog was 1 of 2 dogs included in the study and the other was 1 of 3. None of the other tested dogs from those multidog households were seropositive for IAV. 

## Conclusions 

We detected antibodies to H5 and N1 only in hunting dogs with high levels of bird hunting and waterfowl retrieval. Although that finding suggests transmission of HPIAV H5N1 from waterfowl to dogs can occur, low seroprevalence, lack of reported disease in seropositive dogs, and lack of evidence for dog-to-dog transmission among dogs sharing households collectively indicate that the subclade 2.3.4.4b HPIAV H5N1 strains that circulated in North America during 2022–2023 were poorly adapted to dogs. Those results suggest that effective risk communication with hunting dog owners could be an inexpensive and effective strategy to reduce the potential for spillover to dogs, and monitoring hunting dogs for IAV could be a useful addition to existing surveillance efforts. 

Appendix Additional information about a study of antibodies to influenza A(H5N1) virus in hunting dogs retrieving wild fowl, Washington, USA.
