# Supplementary material for: Antibodies to Influenza A(H5N1) Virus in Hunting Dogs Retrieving Wild Fowl, Washington, USA
Source: Emerg Infect Dis. 2024 Jun;30(6):1271–4. doi: 10.3201/eid3006.231459 (PMC11138995; doi:10.3201/eid3006.231459)
Supplement: Appendix — Additional information about a study of antibodies to influenza A(H5N1) virus in hunting dogs retrieving wild fowl, Washington, USA. [file 23-1459-Techapp-s1.pdf]

Article DOI: https://doi.org/10.3201/eid3006.231459

EID cannot ensure accessibility for Appendix materials supplied by authors. Readers who have difficulty accessing Appendix content should contact the authors for assistance.

Antibodies to Influenza A(H5N1) Virus in Hunting Dogs Retrieving Wild Fowl, Washington, USA

Appendix

Appendix Table 1. The breed composition of bird hunting dogs from Washington state, USA, tested for antibodies to H5 and N1 subtypes of influenza A virus during Spring, 2023.

Dog breed	H5 and N1 seroprevalence (samples seropositive/tested)
Labrador retriever	2/78
Golden retriever	0/40
German shorthaired pointer	0/33
Pudelpointer	0/11
Chesapeake bay retriever	0/9
German wirehaired pointer	1/5
Brittany spaniel	0/4
Germain longhaired pointer	0/3
English setter	0/2
Weimaraner	0/2
Flat-coated retriever	0/2
Boykin spaniel	0/1
Epagneul pont-audemer	0/1
Irish water spaniel	0/1
Small munsterlander	0/1
Standard poodle	1/1
Total	4/194

Appendix Table 2. Frequency of reported bird retrieval activity for hunting dogs (n = 194) in Washington state, USA, during 2022–2023 tested for antibodies to influenza A virus during Spring, 2023.

Frequency of bird retrieval activities	Bird hunting, %	Hunt testing and training with birds, %
0–5	41	10
6–10	11	10
11–15	10	6
16–20	12	6
>20	26	68

Appendix Table 3. Percentage of birds by category reportedly retrieved by hunting dogs (n = 194) from Washington state, USA, during 2022–2023, tested for antibodies to influenza A virus during Spring, 2023. Birds were retrieved by dogs during hunting or hunt testing and training.

Bird category	Hunting dogs reporting retrieval, %
Dabbling ducks	81
Pheasants	62
Pigeons	57
Geese	32
Quail	24
Diving ducks	23
Partridge	14
Doves	10
Grouse	9
Coots	5
Sea ducks	4
